# COVID-19 and the Use of Angiotensin II Receptor Blockers in Older Chronic Hypertensive Patients: Systematic Review and Meta-Analysis

**DOI:** 10.3390/medicina59071200

**Published:** 2023-06-26

**Authors:** Miguel Quesada-Caballero, Ana Carmona-García, Sara Chami-Peña, Luis Albendín-García, Cristina Membrive-Jiménez, José L. Romero-Béjar, Guillermo A. Cañadas-De la Fuente

**Affiliations:** 1 La Caleta Healthcare Unit, Granada-Metropolitan Health District, Andalusian Health Service, 18014 Granada, Spain; miguel.quesada.caballero.sspa@juntadeandalucia.es; 2 Primary Care Emergency Service, Granada-Metropolitan Health District, Andalusian Health Service, 18013 Granada, Spain; ana.carmona.sspa@juntadeandalucia.es; 3 Serranía de Ronda Hospital, AGS ‘Serranía de Málaga’, Andalusian Health Service, 29400 Ronda, Spain; sara.chami.sspa@juntadeandalucia.es; 4 Casería de Montijo Health Center, Granada Metropolitan District, Andalusian Health Service, 18015 Granada, Spain; luis.albendin.sspa@juntadeandalucia.es; 5 Unidad de Farmacogenetica, Servicio de Farmacia Hospitalaria, Hospital Universitario Virgen de las Nieves, Av. de las Fuerzas Armadas, 2, 18014 Granada, Spain; cristina.membrive95@gmail.com; 6 Department of Statistics and Operations Research, University of Granada, 18071 Granada, Spain; 7 Instituto de Investigación Biosanitaria (ibs. GRANADA), 18012 Granada, Spain; 8 Institute of Mathematics, University of Granada (IMAG), 18011 Granada, Spain; 9 Faculty of Health Sciences, University of Granada, 18016 Granada, Spain; gacf@ugr.es; 10 Brain, Mind and Behaviour Research Center (CIMCYC), University of Granada, 18071 Granada, Spain

**Keywords:** angiotensin-converting enzyme inhibitors, ACEI, COVID-19 disease, antihypertensive drugs, older, SARS-CoV-2 infection, selective angiotensin II receptor antagonists, ARAII

## Abstract

Angiotensin II-converting enzyme inhibitors (ACEIs) and selective angiotensin II receptor antagonists (ARAIIs) are widely used antihypertensive agents. Their use has generated controversy due to their possible influence on the health status of chronic patients infected with COVID-19. The objective of this work is to analyze the influence of COVID-19 on chronic hypertensive patients treated with ACEI and ARAII inhibitors. A systematic review and meta-analysis in the databases Pubmed, Pro-Quest and Scopus were carried out. The systematic review was conducted in accordance with the Preferred Reporting Items for Systematic Reviews and Meta-Analyses (PRISMA) guidelines. The search equation descriptors were obtained from the Medical Subject Headings (MeSH) thesaurus. The search equation was: “Older AND hypertension AND (COVID-19 OR coronavirus) AND primary care” and its equivalent in Spanish. Nineteen articles were obtained, with n = 10,806,159 subjects. Several studies describe the COVID-19 association with ACEI or ARAII treatment in hypertension patients as a protective factor, some as a risk factor, and others without a risk association. In the case of ACEI vs. ARAII, the risk described for the former has an odds ratio (OR) of 0.55, and for ARAII, an OR of 0.59. Some authors talk about mortality associated with COVID-19 and ACEI with a half ratio (HR) of 0.97, and also associated ARAIIs with an HR of 0.98. It is recommended to maintain the use of the renin–angiotensin–aldosterone axis in the context of the COVID-19 disease.

## 1. Introduction

A chronic patient is defined as one who has suffered from a non-complicated disease for at least six months. These are the main causes of death and disabilities around the world. The most common chronic diseases are cardiovascular diseases, cancer, chronic obstructive pulmonary disease and diabetes [1]. In the period 2000–2019, 35 million people died, on average, per year due to chronic diseases. Half were under 70 years old and women [2]. For example, deaths from diabetes increased by 70% worldwide between 2000 and 2019, with 80% due to this cause among men [3]. However, despite the high prevalence of all these diseases, heart disease stands out for its high morbidity and mortality [4,5].

Heart disease has been the leading cause of death worldwide for 20 years. The number of deaths due to heart disease has increased since 2000 by more than 2 million people, reaching almost 9 million people in 2019. Heart disease currently accounts for 16% of all deaths among all causes [5,6]. Arterial hypertension remains the most prevalent cause of cardiovascular morbidity and mortality [7]. Several authors confirm AHT as a risk factor. In fact, they have observed in their research that reducing systolic and diastolic blood pressure in hypertensive patients by 5 mmHg reduces cardiovascular events by 10%, cerebrovascular events by 15% and mortality by 5% [8,9].

Notably the clinical data published to date indicate that the most common comorbidity of coronavirus disease 2019 (COVID-19) is hypertension (31.2%), and this is even higher (58.3%) in severe COVID-19 [10]. As guidelines have assessed, renin–angiotensin system blockers (angiotensin-converting enzyme or ACEI/angiotensin II receptor antagonists such as, ARAII) are recommended in the first step of pharmacological treatment. This is supported by the strong evidence of cardiovascular and renal protection with these drug classes [11]. Therefore, these antihypertensives have a theoretical increase in COVID-19 mortality because of their inducible effect on the expression of angiotensin-converting enzyme II (ACEII), the known functional receptor utilized by severe acute respiratory syndrome coronavirus 2 (SARS-CoV-2) [12].

The COVID-19 disease appeared at the end of 2019 in Wuhan (China), caused by a new coronavirus called SARS-CoV-2, generating the declaration of a sanitary alert and pandemic in the first quarter of 2020 [13]. This virus has caused 553 million cases and 6.3 million deaths worldwide by July 2022 [14]. SARS-CoV-2 is a ribonucleic acid (RNA) virus, of which the activity depends on its cellular introduction for the replication of its genetic material, which is produced using angiotensin-converting enzyme II (ACEII) as a receptor by interacting with the spike protein (S) of Coronavirus SARS-CoV-2 mediated by the transmembrane protease serine 2 (TMPRSS2) [15,16]. Some research speculated that drugs inhibiting the renin–angiotensin–aldosterone system might upregulate SARS-CoV-2 cell receptors, thereby aiding viral replication [17].

The clinical picture is variable, and it is estimated that up to 33% of patients are asymptomatic [18]. Most patients have a controlled infection that can occur with or without pneumonia, but responds well to home treatment. However, 14% present with severe dyspnea and hypoxia and/or pneumonia that affects more than 50% of the lung field in 24–48 h, with a severe course and hospital admission. Multi-organ failure mediated by a cytokine storm is developed by 5%, that requires admission to intensive care units (ICUs) [19]. Of the cases, 2.3% die from complications arising during the COVID-19 process [20]. This is a common occurrence, as several studies have revealed that individuals with heightened cardiovascular risk (e.g., high blood pressure) have a greater tendency to experience severe outcomes due to COVID-19 [21].

COVID-19 has a higher morbidity and mortality in patients with chronic diseases, including hypertensive patients, among many others [22]. There is evidence of greater severity and mortality with older age, being more accentuated in those older than 65 years [23]. All these patients are more likely to have sequels, defined as persistent COVID, where the patient has symptoms of the disease for more than two months after the end of the infection, and of which the appearance is more likely the more severe the infection has been [24]. In fact, the risk of adverse cardiovascular events in post-COVID patients is increased up to one year after infection, acting as another risk factor [25].

ACEIs/ARAIIs are the most widely used antihypertensive drugs in clinical practice. There are articles with different points of view, variably supporting their use in patients affected by COVID-19. The review of the subject proposed in this manuscript serves to check their safety in patients and evaluate if other treatment alternatives are also safe.

Therefore, the aim of this study was to analyze the relationship between SASR-COV2 and antihypertensive treatment with ACEI or ARAII in the older adults using a protocoled systematic review methodology. Different antihypertensive drugs were used to observe existing data and their possible comparison.

## 2. Materials and Methods

### 2.1. Design

This work was conducted according to the Preferred Reporting Items for Systematic Reviews and Meta-Analyses (PRISMA) guidelines [26]. The study was registered (ID: 354488) in the PROSPERO database (International Prospective Register of Systematic Reviews).

### 2.2. Information Sources and Search Strategy

The following databases were consulted: Pubmed, Pro-Quest and Scopus. The MeSH terms employed in the search strategy were “Older AND hypertension AND (COVID-19 OR coronavirus) AND primary care”, and their equivalent in Spanish. The search was conducted in August 2022.

### 2.3. Eligibility Criteria

Inclusion criteria: quantitative primary studies analyzing the relationship between ACEI and ARAII, and the pathology of COVID respiratory syndrome in patients over 60 years of age; published in English and/or Spanish; without restriction by year of publication.

Exclusion criteria: doctoral theses, articles without statistical information, duplicate studies, those not carried out in adults, or of which the main objective was not to investigate the relationship between ACEI/ARAII drugs and their relationship with the disease developed by the SARS-CoV-2 coronavirus.

### 2.4. Study Selection Process

Two members of the team (M.Q.-C. and C.M.-J) performed the search and study selection independently. In case of a disagreement, a third researcher (A.C.-G.) was consulted. The selection of articles was carried out in four steps: (1) reading the title and abstract, (2) discarding those that did not meet the inclusion criteria, (3) reading the full text, and (4) a reverse search.

### 2.5. Data Collection and Synthesis

To extract the data from each study, a data collection notebook was created that includes the first author, year of publication, country of the study, design, sample, intervention on which the article has based its study, means with their standard deviation, main results, and level of evidence and grade of the recommendation.

### 2.6. Risk of Bias Assessment and Level of Evidence

Risk of bias was performed using a Cochrane risk of bias assessment for clinical trials and the elimination questions from the CASP (Critical Apprais-al Skills Program) for cohort studies. The quality of the studies included in this review was assessed following the levels of evidence and grades of recommendation stipulated by the Oxford Center for Evidence-Based Medicine (OCEBM) [27].

### 2.7. Data Analysis

A descriptive analysis of the information from the included studies was performed for the systematic review. A fixed effects meta-analysis using Review Manager 5.4 was performed. The effect size (odds ratio) for all-cause mortality with the continuation or discontinuation in the ACE inhibitor treatment was calculated. I^2^ was calculated for heterogeneity.

## 3. Results

### 3.1. Study Selection and Study Characteristics of the Studies Included

This review has 18 selected articles from 13 nationalities, including 10,802,367 patients.

**Figure 1:**
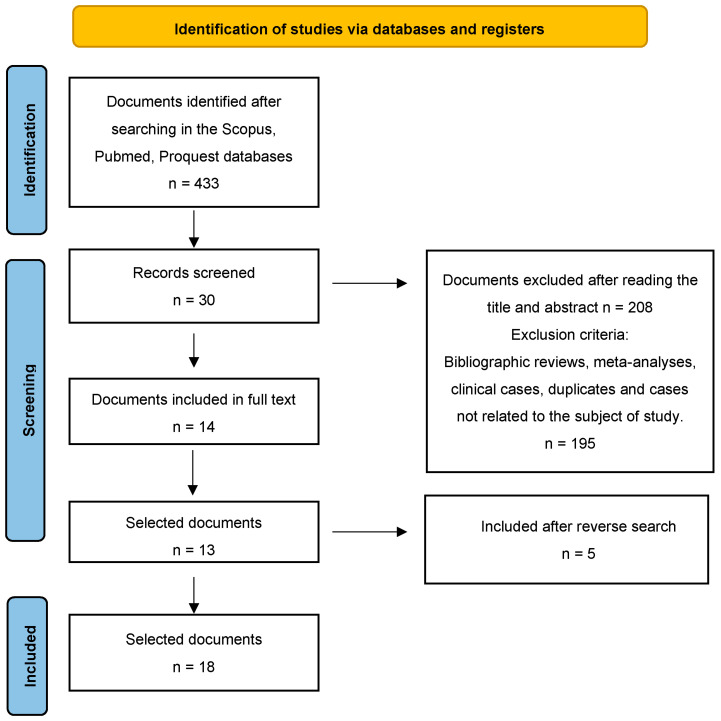
Flow diagram of the publication search process [26].

After conducting the search, 433 studies were found. After reading the title and abstract, 209 articles were selected. Of these manuscripts, 195 were discarded because they were duplicates, were not quantitative studies or were not related to the subject. Of the remaining 14 articles, one was excluded as it was not a full text. Finally, five articles were included after a reverse search. Therefore, the final sample was n = 18 definitive articles for analysis (Figure 1).

**Figure 2:**
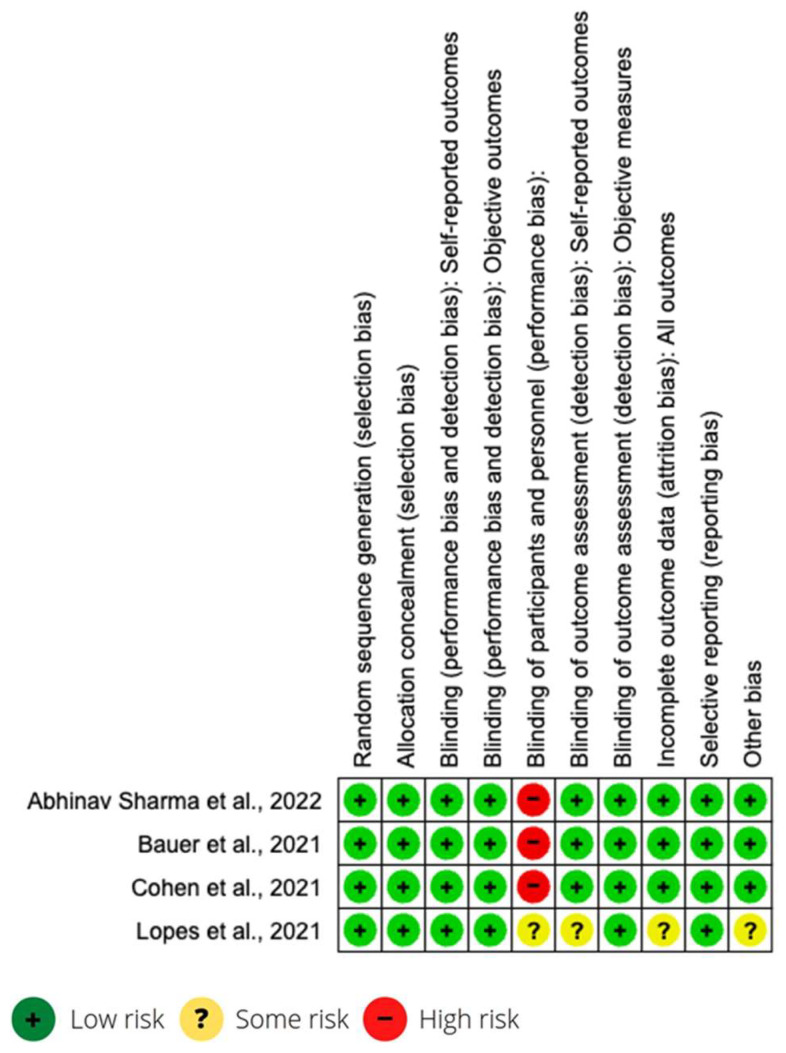
Risk of bias of clinical trials [28,30,36,43].

All studies were longitudinal, and the sample selection was randomized. Most of the studies were carried out in other countries (Belgium, USA, Canada, Mexico, Sweden, Peru, Bolivia, Argentina, Germany, Austria, England, Kuwait, Italy, Brazil and Canada). Only one was carried out in Spain, with a mixed Spain–USA study. The articles correspond to 14 cohort studies, 4 clinical trials and 1 case-control study. Information on the characteristics of each study is found in the first two columns of Table 1. The risk of bias in studies is presented in Figure 2 and Table 2.

**Table 1:** Characteristics of each study with associations of existing antihypertensive treatment in the articles.

Author, Year, Country	Design	Sample/Age/Prevalence	Intervention	Mean ± SD	Measures	Main Results/ ICU Admission	LE/GR
Bauer et al., 2021 (Austria and Germany) [28]	Randomized controlled clinical trial (RCT)	n = 204 outpatients; Age = 75 Continuation Group (1) = 100; Discontinuation Group (2) = 104 Dyslipidaemia = 86 (42%); DMT2 = 67 (33%); Atrial Fibrilation = 35 (17%); Kidney Disease = 37 (18%); IMC = 27.5; HBP = 199 (98%) *Vital Parameters* Beats/mi: (1) 76–(2) 76; Respiratory/min: (1) 18–(2) 18; Temperature: (1) 37–(2) 37; Blood pressure: (1) 130/77–(2) 130/75; Oxygen Saturation: (1) 94%–(2) 95% *Respiratory Therapy* Oxygen substitution: (1) 45–(2) 41 Oxygen mask therapy: (1) 43–(2) 37 Non-invasive ventilation: (1) 5–(2) 0 *Radiological Signs* Bipulmonar infiltrates: (1) 36–(2) 38 Other opacities: (1) 29–(2) 26 C reactive protein: (1) 5.1–(2) 4.5	Comparison ACEI/ARAII Versus not taking them regardless of whether they are hospitalized	N1 = 104 withdraws ACEI/ARA II N2 = 100 continues ACEI/ARA II Max SOFA score 0 (0–2) vs. 1 (0–3) *p* = 0.12 Lower AUCsofa 0 (0–9.25) vs. 3.5 (0–23.5) *p* = 0.040 Mean SOFA score 0 (0–31) vs. 0.12 (0–0.78) *p* = 0.040 30 days SOFA score 0 (0–1.20) vs. 0 (0–24) Patients with severe involvement with mechanical ventilation (10% N1 vs. 8% N2 *p* = 0.87) or in ICU (19% N1 vs. 18% N2 *p* = 0.96) At 30 days, 11% of the withdrawal group and 23% of the drug continuation group had signs of organ dysfunction or have passed away *p* = 0.017.	1—Max SOFA score 2—Lower AUCSOFA 3—Mean SOFA 4—30 days SOFA score Prevalence Severity ICU Mechanical ventilation	Withdrawing ACEI/ARAII in COVID-19 patients had no effect in severely affected patients with mechanical ventilation or in ICU, but the results of this trial indicate that withdrawal of ACEI and ARAII may allow a faster and better recovery. / YES	1a/A
Bean et al., 2020 (England) [29]	Cohorts	n = 1200 patients hospitalized in London Age = 63 +/− 20 SD Heart Disease = 107 (8%); DMT2 = 418 (35%); Kidney Disease = 206 (17%); Obesity = 182 (15%); Stroke = 235 (20%); Chronic Obstructive Lung Disease = 121 (10%); HBP = 645 (53%)	Comparison of ACEI/ARAII versus not taking them in hospitalized patients	N1 (ACEI/ARAII) = 399 N2 (no ACEI/ARAII) = 801 COVID-19 severity results OR 0.63 IC 0.47–0.84 (*p* = 0.01)	Odds Ratio Prevalence Severity	There is no evidence of an increase in the severity of COVID-19 in patients treated with ACEIs and ARBs in the study, and a possible protective factor is appreciated that must be corroborated in subsequent clinical trials. / NO	2a/B
Cohen et al., 2021 (USA, Canada, Mexico, Sweden, Peru, Bolivia, Argentina) [30]	Randomized controlled clinical trial (RCT)	n = 152 hospitalized patients Age = 62 male (52%) and 68 female (48%) Hispanic 82 (54%) DMT2 79 (52%) with insuline 36 (23.7%) HBP = 152	They compare ACEI/ARAII versus not taking them in hospitalized patients	N = 75 patients continue with ACEI/ARAII y N = 77 withdraws. Mean Rank 73 (IQR 40–100) for continuation group and 81 (IQR 38–117) for the group that withdraws. X^2^ for adverse events without differences between both groups *p* = 0.77	Mean Rank X^2^	No differences were found in the GLOBAL RANK SCORE, nor in adverse events, blood pressure, nor in serum levels of potassium or creatinine between both groups. / YES	1a/A
De Spiegeleer et al., 2020 (Belgium) [31]	Cohorts	n = 154 patients with COVID-19 in home care Age = 86 +/− 7 SD COVID symtoms = 113 Asymptoms = 41 DMT2 = 28 (18%) HBP = 39 (25%)	They compare ACEI/ARAII and statins versus not taking them in non-hospitalized patients	Statins and the absence of COVID-19 symptoms (OR 2.91; CI 1.27–6.71) Statins and COVID-19 clinical improvement (OR 0.75; CI 0.24–1.87) ACEI/ARAII and COVID-19 clinical improvement (OR 0.48; CI 0.1–1.97).	Odds Ratio Prevalence	Finds significantly statistical results in taking statins and the absence of symptoms during COVID-19 infection. / NO	2a/B
Elabd et al., 2021 (Kuwait) [32]	Cohorts	n = 4019 hospitalized in Kuwait City Age = 43.49 Heart Disease = 168 (4%); DMT2 = 634 (15%); Chronic Obstructive Lung Disease = 17 (0.4%); HBP = 782 (20%)	They compare ACEI/ARAII versus not taking them in hospitalized patients	N1 (ACEI/ARAII) = 325 N2 (no ACEI/ARAII) = 3694 N1 is inversely associated with ICU admission OR 0.57 CI 0.34–0.88 (*p* = 0.01) and inversely associated with mortality OR 0.56 IC 0.33–0.95 (*p* = 0.032).	Odds Ratio Prevalence Mortality	The results recommend continuing ACEI/ARAII in patients who acquire COVID-19. The protective effects of the study support this hypothesis. / YES	2a/B
Fosbϕl et al., 2020 (Denmark) [33]	Cohorts retrospectives	n = 4480 patients without discriminating hospitalized or not. Age = 54.6 Heart Disease = 243 (5%); Atrial Fibrillation = 317 (7%); DMT2 = 411 (9%); Kidney Disease = 172 (4%); Stroke = 402 (9%); Chronic Obstructive Lung Disease = 634 (14%); HBP = 843 (18%)	They compare ACEI and ARAII versus not taking them regardless of being hospitalized.	N1 (ACEI/ARAII) = 895 N2 (no ACEI/ARAII) = 3585 Mortality 0.83 HR IC 0.67–1.03 *p* = 0.05 Death in severe COVID-19 HR 1.04 IC 0.89–1.29 *p* = 0.05 Incidence of COVID-19 in N1 HR 1.05 IC 0.80–1.36 *p* = 0.05	Hazard Ratio Incidence Mortality Severity	ACEI/ARAII is not significantly associated with the diagnosis of COVID-19, nor with the severity of the infection nor with increased mortality. / NO	2a/B
Hippisley-Cox et al., 2020 (England) [34]	Cohorts retrospectives	n = 8,275,949 Age = 48.47 PV = 19,486 (0.2%) COVID patients without discriminating hospitalization (1286 ICU of them) Heart Disease = 433,631 (5%); Atrial Fibrillation = 201,911 (2.4%); DMT2 = 536,516 (6%); Kidney Disease = 338,693 (4%); Obesity = 1,709,529 (20%); Chronic Obstructive Lung Disease = 195,115 (2%); HBP = 1,414,021 (17%)	They compare ACEI and ARAII versus not taking them regardless of being hospitalized.	N1 = ACEI = 2864 N2 = ARAII = 1417 N2 = no ACEI/ARAII = 3304 N1 vs. N3 COVID-19 risk HR 0.71 IC 0.67–0.74 *p* = 0.05 N2 vs. N3 COVID-19 risk HR 0.63 IC 0.59–0.67 *p* = 0.05 N1 vs. N3 increase ICU risk HR 0.89 IC 0.75–1.06 *p* = 0.05 N2 vs. N3 increase ICU risk HR 1.02 IC 0.83–1.25 *p* = 0.05	Hazard Ratio Prevalence Severity	ACEI and ARAII are significantly associated with a reduced risk of COVID-19 in this study. / YES	2a/B
Jeffery et al., 2022 (USA) [35]	Cohorts retrospectives	n = 1,059,474 Age = 72,623 Heart Disease = 254,773 (24%); DMT2 = 422,780 (40%); Kidney Disease = 212,362 (20%); Obesity = 116,557 (11%); Stroke = 73,361 (7%); Chronic Obstructive Lung Disease = 372,735 (35%); HBP = 1,059,474 (100%)	Reported association between ACEI/ARAII use and respiratory viral diseases without discriminating in-hospital and out-of-hospital patients.	N1 = 653,797 IECA/ARAII N2 = 405,677 No IECA/ARAII ICU risk 1.5 pp IC (1.2–1.9) (*p* = 0.05) Dyspnea Risk 0.7 pp (0.1–1.2 IC) (*p* = 0.05) AVDS Risk 0.9 pp (0.4–1.3 IC) (*p* = 0.05)	Percentage Point	Patients with AVRIs using ACEi/ARAII for HTN had a greater increase in poor outcomes during the COVID-19 pandemic than those using other HTN drugs. / YES	2a/B
Lopes et al. 2021 (Brazil) [36]	Randomized controlled clinical trial (RCT)	n = 659 Age = 55.5 (46.1–66.1) Heart Disease = 39 (5%); DMT2 = 210 (32%); Kidney Disease = 9 (1%); Obesity = 341 (52%); HBP = 659 (100%)	Determine whether discontinuation compared with continuation of ACEI or ARAII changed the number of days alive and out of hospital within 30 days.	N1 Discontinue IECA/ARAII = 334 N2 Continue IECA/ARAII = 325 Not statistical association for Death Cardiovascular Death Evolution Days alive Day salive out of hospital *p* = 0.3	Odds Ratio	In patients hospitalized with mild-to-moderate COVID-19 and who were taking ACE inhibitors or ARBs prior to hospital admission, there was no significant difference in the mean number of days alive and out of hospital for those assigned to Discontinue vs. continue these medications. / NO	1a/A
Mancia et al., 2020 (Italy) [37]	Control cases	n = 37,031 Age = 68 +/− 13 SD VP = 6272 (16.93%) C = 30,759 Heart Disease = 8570 (23%); Kidney Disease = 1129 (3%); Chronic Obstructive Lung Disease = 521 (1%); Cancer = 5729 (15%)	They compare ACEI and ARAII versus not taking it and with other antihypertensive regardless of being hospitalized or not	N1 = ACEI 1508 N2 = ARAII 1394 N3 = ACC 1446 N4 = DIURETICS 1902 OR N1 COVID-19 = 0.95 (0.86–1.05) *p* = 0.05 OR N2 COVID-19 = 0.96 (0.87–1.07) *p* = 0.05 OR N1 Severity/Lethality 0.83 (0.63–1.10) *p* = 0.05 OR N2 0.91 (0.69–1.21) *p* = 0.05	Odds Ratio Severity Mortality	There is no evidence of an association between ACEI and ARA II and the risk of COVID-19 or increased severity or lethality / NO	2b/B
Mazzoni et al., 2022 (Italy) [38]	Cohorts retrospectives	n = 615 Age = 70.9 Atrial Fibrillation = 54 (9%); DMT2 = 107 (17%); Kidney Disease = 15 (2%); Obesity = 38 (6%); Chronic Obstructive Lung Disease = 25 (4%); Heart Disease = 59 (10%); ICU = 96 (15,6%); HBP = 86 (14%)	Analyzes hospitalized patients in one area of Italy and compares deaths who have taken ACEI/ARAII with Haven taken other hypertensive drug to observe a possible association.	N1 IECA/ARAII Death 86 N2 no IECA/ARAII Death 78 30 days alive 94.9% N1 91.8 N2 Mortality 2.8% N1 2.7% N2 IC (0.3–2.52) (*p* = 0.03)	Odds Ratio	The apparent increase in morbidity in patients with COVID-19 who received long-term treatment with ACE inhibitors or ARBs is not due to the drugs themselves, but to the conditions associated with their use. / NO	2a/B
Morales et al., 2021 (Spain and USA) [39]	Cohorts	n = 1,355,349 hypertensive patients using ACE inhibitors/ARBs (363,785 monotherapy) Age = 67.20 Data from: SIDIAP 37796 (System for Research in Primary care) (1) VA-OMOP 320450 (Veterans Affair Observational Medicals outcomes) (2) CUIMC 5539 (Columbia University Irving medical Center) (3) *Adjusted dates for Analysis* Dyslipidemia: (1) 27%–(2) 44.7%–(3) 35.5%; DMT2: (1) 20.7%–(2) 29.5%–(3) 16.7%; Heart Disease: (1) 21.8%–(2) 13.3%–(3) 26.4%; Atrial Fibrillation: (1) 4.1%–(2) 2.7%–(3) 5%; Kidney disease: (1) 9.4%–(2) 6.7%–(3) 7.8%; Obesity: (1) 35.5%–(2) 11.9%–(3) 9.3%; Stroke/Cerebrovascular: (1) 2.2%–(2) 2.3%–(3) 5.2%; Chronic Obstructive Lung: (1) 6.2%–(2) 8.4%–(3) 2.9%; HBP: (1) 99.2%–(2) 68%–(3) 61%	They compare ACEI and ARAII versus not taking them regardless of being hospitalized.	N1 (ACEI/ARAII in monotherapy) = 363,785 N2 (ACC/HCLTZ in monotherapy) = 248,915 N3 (ACEI/ARAII in combination) = 711,799 N4 (ACC/HCLTZ in combination) = 473,076 Risk N1 vs. Risk N2 HR 0.98 IC 0.84–1.14 *p* < 0.05 Risk N3 vs. Risk N4 HR 1.01 IC 0.9–1.15 *p* < 0.05 Risk ACEI vs. Risk N2 HR 0.91 IC 0.68–1.21 *p* < 0.05 Risk ACEI vs. Risk N4 HR 0.95 IC 0.83–1.07 *p* < 0.05 Risk ACEI vs. Risk ARAII (both in combination) HR 0.88 IC 0.79–0.99 *p* < 0.05 Risk ACEI vs. risk ARAII (monotherapy) (HR 0.85 IC 0.69–1.05)	Relative risk	There is no significant increase in the risk of diagnosis of COVID-19 or in the results associated with ACEI/ARAII. / NO	2a/B
Palazzuoli et al., 2020 (Italy) [40]	Cohorts retrospetives	n = 781 patients hospitalized for COVID-19 Age = 69 Heart Disease = 171 (21%); DMT2 = 143 (18%); Chronic Obstructive Lung Disease = 84 (11%); BP = 451 (58%); ICU care = 225 (29%)	They compare ACEI/ARAII versus not in hospital for patients over 50 years old.	N1 (ARAII) = 131 N2 (ACEI) = 171 N3 (no ACEI/ARAII) = 477 Mortality N1 OR 0.58 IC 0.35–1.07 *p* = 0.0796 Mortality N2 OR 0.55 IC 0.3–0.98 *p* = 0.0436	Odds Ratio Prevalence Mortality	In patients over 50 years of age hospitalized for COVID-19, the use of ACEIs significantly reduces the risk of death. / NO	2a/B
Peñalvo et al., 2021 (Belgium) [41]	Cohorts	n = 10,866 hospitalized patients from 119 Belgian hospitals Age = 67.82 Heart Disease (CVD) = 3984 (37%); DMT2 = 2522 (23%); Kidney Disease = 1513 (14%); Obesity = 782 (7%); Chronic obstructive Lung Disease = 1731 (16%); HBP = 4593 (42%); Cognitive issues = 1320 (12%); ICU care: (1) 425–(2) 990; Length of hospital stay: (1) 13.9–(2) 12.1	They compare ACEI/ARAII versus not taking them in hospitalized patients	ACEI/ARAII in non-ICU patients are associated with a slight increase in recovery HR 1.07 IC 1.01–1.13 (*p* = 0.027) and mortality reduction HR 0.83 IC 0.75–0.93 (*p* = 0.001) not so in ICU patients in recovery HR 1.16 IC 0.97–1.38 (*p* = 0.098) nor in reduction of ICU mortality HR 0.91 IC 0.73–1.12 (*p* = 0.381)	Hazard Ratio Prevalence Recovery Mortality	The use of ACEI/ARAII in hospitalized patients, according to the results observed, protects the patient not admitted to the ICU, being associated with a discreet reduction in mortality. / YES	2a/B
Sha et al., 2020 (USA) [42]	Cohorts retrospetives	n = 531 Afro-american patients hospitalized for COVID-19 Age = 60.01 +/− 15 Congestive Heart Disease = 79 (14%); DMT2 = 228 (42%); Kidney disease = 77 (14%); Chronic Obstructive Lung Disease = 36 (6%); BMI = 35 +/− 8.1; HBP = 425 (80%)	Comparison of ACEI/ARAII versus not taking them in hospitalized Afro-Americans	N1 (ACEI/ARAII) = 207 N2 (no ACEI/ARAII) =324 Hospital Mortality 18,4% N1 vs. 14,8% N2 (*p* = 0.28) Mechanical ventilation 22.2% N1 vs. 16% N2 (*p* = 0.07) Hospital length of stay 10 days N1 vs. 8.8 days N2 (*p* = 0.14)	Relative risk	The use of ACEI/ARAII in hospitalized Afro-Americans in the study carried out does not show differences with respect to withdrawing them. / YES	2a/B
Sharma et al., 2022 (Canada) [43]	Randomized controlled clinical trial (RCT)	n = 46 Age = 69 Dislypidaemia = 27 (59%); Heart disease = 15 (32%); Atrial Fibrillation = 7 (15%); DMT2 = 20 (43%); Kidney Disease = 9 (20%); HBP = 46 (100%); Stroke = 3 (6%); Chronic Obstructive Lung Disease = 2 (4%)	Compares continue ACEI/ARAII treatment versus Discontinuation ACEI/ARAII. Hospital Study	N1 continue IECA/ARA II = 21 N2 discontinue IECA/ARAII = 25 Results p greater than 0.05 not statistically significant for BNP increase, heart failure and risk of adverse outcome.	Standard deviation	The continuation of RAASi in hospitalized participants with COVID-19 appears safe. It cannot be associated with the data increased risk of COVID-19 disease or morbidity and mortality to ACEi/ARAII. / YES	1a/A
Trifiró et al., 2020 (Italy) [44]	Cohorts retrospectives	n = 42,926 hospitalized patients Age = 69 Heart Disease = 10,019 (23%); Atrial Fibrillation = 2899 (7%); DMT2 = 7710 (18%); Kidney Disease = 1046 (2%); Stroke = 3441 (8%); Chronic Obstructive Lung Disease = 1521 (4%); HBP = 5610 (13%); Death = 11,205 (26%)	They compare ACEI/ARAII against calcium antagonists and against no antihypertensive in hospitalized patients	N1 = ACEI 4663 N2 = ARA II 4859 N3 = ACC 2178 N4 no ATH treatment = 21,974 N5 Other antihypertensive = 4068 Death risk N1 HR 0.97 IC 0.89–1.06 *p* = 0.05 Death risk N2 HR 0.98 IC 0.89–1.06 *p* = 0.05 Compared both with ACC	Hazard Ratio Mortality	ACEI and ARA II are not associated with an increased or decreased risk of mortality compared to ACC. / YES	2a/B
Zhang et al., 2020 (China) [45]	Cohorts retrospectives	n = 3611; PV = 1128 (31.23%); HBP = 525 (47%); Age = 64; HBP = 1128 (31%); Heart Disease in HBP = 131 (12%); DMT2 in HBP = 200 (18%); Stroke in HBP = 41 (3%); Chronic Obstructive Lung in HBP = 6 (0.5%)	They compare ACEI and ARAII versus not taking them regardless of being hospitalized	N1 = ACEI/ARAII = 188 N2 = no ACEI/ARAII = 940 Mortality N1 vs. N2 HR = 0.42 (0.19–0.92) *p* = 0.03 COVID-19 Mortality Risk N1 vs. N2 HR = 0.37 (0.15–0.89) *p* = 0.03. Comparison of other antihypertensive drugs Mortality N1 HR = 0.3 (0.12–0.70) *p* = 0.01	Hazard Ratio Mortality	ACEI/ARAII in patients hospitalized for COVID-19 with hypertension is associated with lower mortality than all causes and from COVID-19. / NO	2a/B

Remark: ACEI: angiotensin-converting enzyme inhibitors; ARAII: angiotensin II receptor antagonist; ARBII: angiotensin II receptor blocker; ARDS: acute respiratory distress syndrome; BB: beta-blocker; BMI: Body Mass Index; CCB: calcium channel blocker; CID: coagulation intravascular disseminate; CVD: cardiovascular disease; DMT2: diabetes mellitus type 2; GR: grade of recommendation; HBP: high blood pressure; IQRs: interquartile ranges; ICU: intensive care unit; LE: level of evidence; RAASi: renin angiotensin aldosterone inhibitors; RCT: randomized controlled clinical trial; SD: standard deviation; SOFA: sequential organ failure assessment score.

The number of patients studied (N) is 10,802,367, of which 8,280,000 correspond to the study of Hippisley-Cox et al. [34], 1,355,349 to that of Morales et al. [39] and 1,059,474 to Jeffery MM. et al. 2022, that are the three articles that exceed one million patients [25,26,27,28,29,30,31,32,33,34,37,39,40,41,42,44,45].

The mean age of the articles ranged from 48.41 to 86 +/− 7 years. The prevalence of diabetes, hypertension and obesity as the main comorbidities in COVID-19 patients was collected. Data were also collected on renal failure, COPD, heart failure and atrial fibrillation, as well as stroke, as the diseases that most frequently alter blood pressure figures. These figures and the associations of existing antihypertensive treatment in the articles are shown in Table 1.

### 3.2. Risk Association to the Use of ACEI/ARAII

There are seven studies discussing protective factors, one discussing risk factors and nine without risk associations.

According to the criteria for defining the risk associated with ACEI/ARAII, the articles studied are classified into three groups. First, the papers [29,32,34,39,40,41,45] establish the drugs studied as a protective factor or decrease of severity and fatality conditions. Zhang et al. [45], however, found a decrease in mortality caused by all causes in COVID patients. Elabd et al. [32] observed decreased ICU admission and increased recovery in ACEI and ARAII patients. Pallazuoli et al. [40] show decrease of COVID-19 risk in patients with ACEI treatment. Morales et al. [39] analyzed less COVID-19 risk in ACEI patients, and Hippisley-Cox et al. [34] found less COVID-19 risks in ACEI/ARAII patients. Peñalvo et al. [41], as well as Bean et al. [29] deduced no increasing severity and a slight protective factor that requires more evaluation. The second group [30,35,36,37,43,45,46] did not establish a risk or protection association with respect to the consumption of ACEI/ARAII. Finally, conversely to the studies above, Bauer et al. [28] reported that when studying patients with severe involvement, an increase or reduction in risk cannot be observed. However, they indicate that the results point to a faster and better recovery of patients if ACEI/ARAII is withdrawn from COVID patients [31]. They also advise of a worsening outcome of COVID-19 respiratory syndrome in patients using ACEI/ARAII than in the rest of the hypertensive patients treated with other medication. Nevertheless, there are slight differences that do not allow recommending the withdrawal of treatment in these patients [33].

### 3.3. Risk Association to the Use of ACEI vs. ARAII

Six studies show the comparation of ACEI to ARAII.

Four of the articles addressed a statistical comparison of the results between these drugs. In Palazzuoli et al. [40], a statistical study of mortality was carried out where an association with ACEI (OR 0.55) and ARAII (OR 0.59) was established. They found a significant protective factor on the part of both. Trifiró et al. [44] study the mortality risk of COVID associated with ACEI (HR 0.97) and ARAII (HR 0.98), not observing significant data. Mancia et al. [37] postulate an association between the risk of COVID and the lethality/severity of the disease associated with the consumption of ACEI/ARAII, with an OR of 0.95 for risk associated with ACEI and 0.96 for ARAII. An OR of 0.83 was found in lethality/severity associated with ACE inhibitors and OR 0.91 in severity/lethality associated with ARAII. Hippisley-Cox et al. [34] associated the use of ACEI and ARAII with the risk of COVID and the risk of admission to the ICU: HR 0.71 (0.67–0.74) for ACEI and the risk of COVID, HR 0.63 (0.54–0.67) for ARAII and the risk of COVID, HR 0.89 (0.75–1.06) for ACEI and ICU risk, and HR 1.02 (0.83–1.25) for ARAII and ICU risk.

Morales et al. [39] observed an ACEI-associated significantly lower risk of COVID-19 in combination use (HR 0.88 (0.79–0.99)).

### 3.4. Risk Association to the Use of ACEI/ARAII vs. Calcium Antagonists (CA) in COVID

Four articles analyze ACEI/ARAII vs. CA in COVID.

Morales et al. [39] did not observe a torpid COVID-19 evolution or COVID-19 hospital admission increase. Pneumonic risk results with CA, ACEIs and ARAII were similar in monotherapy (HR 0.98) or in combination (HR 1.01). Trifiró et al. [44] did not appreciate an increase or decrease in mortality among patients using ACEI (HR 0.97) or ARAII (HR 0.98) compared to CA treatment (HR 1.02 + ACEI) (HR 1.05 + ARAII) or in monotherapy (HR 1.11). Mancia et al. [37] associated CA as a risk factor (OR 1.03) versus ACEI (OR 0.95) ARAs (OR 0.96) as a COVID-19 infection and mortality protection factor. In addition, they reported a combination of antihypertensive drugs with ACEI (OR 0.99) or ARAII (OR 0.98) compared to monotherapy (OR 1.03). Hippisley-Cox et al. [34] found statistical association of COVID-19 risk (HR 0.92) and the risk of developing severe COVID-19 and ICU admission (HR 1.33). ACEI decreased both risks (HR 0.71) (HR 0.89) and ARA II decreased only the COVID-19 infection risk (HR 0.63).

### 3.5. Risk Association to the Use of ACEI/ARAII vs. Diuretics in COVID

Three studies were conducted with ACEI/ARAII vs. diuretics in COVID.

Mancia et al. [37] described ASA diuretics as a risk COVID-19 factor (OR 1.46) and did not observe association in treatments with thiazide diuretics (OR 1.03). Hippisley-Cox et al. [34] did not find an association between potassium-sparing diuretics and COVID-19 disease (HR 1.03), and reported thiazides as a COVID-19 protecting factor (HR 0.70). Neither of the two diuretics obtained significant data associated with ICU admission (HR 0.6) (HR 0.9).

Morales et al. [39] did not observe an association between COVID-19 diagnosis and exposure to ACEI/ARAII versus thiazide diuretics monotherapy (HR 0.98 (0.84–1.14)) and combination (HR 1.01).

### 3.6. ACEI/ARAII Complications in COVID

The data of high mortality in ACE + ARAII are also used in COVID.

According to several authors, the combined use (ACEI + ARAII) has an excessive risk of hyperkalemia, hypotension and renal failure, and a high mortality rate compared to the use of monotherapy. These data are applicable to COVID patients [36]. Bauer et al. [28] indicated a possibly faster and more complete recovery for suspended ACEI/ARAII therapy, contrary to Peñalvo et al. who affirmed a faster recovery by continuing ACEI/ARAII [28].

### 3.7. Meta-Analysis of ACEI

Of the studies included in the systematic review, only two included the data necessary for a meta-analysis of the effect size.

**Figure 3:**
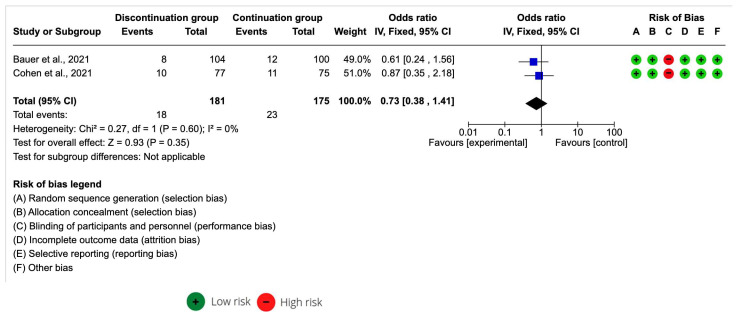
Forest plot of all-cause mortality after the continuation and discontinuation of ACEI [28,30].

The ACEI discontinuation group sample was n = 181 and the ACEI continuation group was n = 175. The meta-analysis estimation for an all-cause mortality odds ratio was 0.73 [95% CI 0.38–1.41] with *p* > 0.05. I^2^ was 0% (Figure 3). A decrease in all-cause mortality in COVID in Bauer et al. [28] and Cohen et al. [30] was associated with discontinued ACEI.

## 4. Discussion

The role of ACEI/ARAII in the framework of COVID-19 disease has been questioned since the beginning of the pandemic. The possibility that they were harmful or beneficial has been raised and is currently being evaluated for the benefit of the patient [36,46].

Angiotensinogen is discharged by the liver. Renin facilitates the conversion of angiotensinogen I into angiotensinogen II, while the angiotensin-converting enzyme (ACE) converts to angiotensinogen II, which exhibits potent vasoconstrictive effects. Angiotensinogen II stimulates the release of aldosterone, which plays a role in retaining water and sodium in the human body. SARS-CoV-2 enters cells by binding to ACE, potentially modifying its function. In fact, studies indicate that there is an elevated level of ACE in older patients with hypertension, which may increase their susceptibility to infection [47].

The data that advocate a beneficial role of ACEI and ARAII are based on the existence of lower levels of cytokines, decreasing inflammatory levels and IL6 expression in patients with severe COVID-19, and lower associated mortality data [48,49,50], as well as a possible protective factors for severe cases [51,52]. However, this has not been demonstrated in clinical practice [53]. A recent clinical trial has not been able to show improvement in lung function in patients with losartan (ARAII) [54] or provide statistically significant data that may have a clinical impact [55]. Heart congestive failure and atrial fibrillation can be an adverse evolution in hypertensive patients, but actually, in the COVID era, hypertensive patients cannot be associated ACEI/ARAII use [56,57]. Recent studies have found that pre-treatment with ACEI/ARAII could be continued, as it was associated with lower hospital mortality, ICU admission and IMV in patients with COVID-19 [58]. For this reason, this medication should not be discontinued, especially ARAIIs [59,60,61].

Initial uncertainty about the role of hypertension and its treatment led to heterogeneous management and major changes in antihypertensive treatment, mainly at the cost of discontinuation of ACE inhibitors or ARAIIs. However, there is now consistent evidence that the use of antihypertensive drugs is not associated with the risk and severity of COVID-19 [17]. Survival with withdrawal of ACEI/ARAII in patients hospitalized in the ward does not improve [43]. In critical patients with hemodynamic repercussion and low blood pressure, its withdrawal can be assessed, but in the rest of the hypertensive patients with ACEI/ARAII and COVID disease, its use continues to be considered in a hospital setting [62]. In fact, changes in antihypertensive treatment are still made in older hospitalized patients due to their comorbidities [63].

Regarding calcium antagonists, the data from our study have not been able to find an association with COVID-19. Other studies claim that any first-line antihypertensive treatment decreases had a significantly lower risk of in-hospital death [64], although it is true that the literature consulted shows a higher risk of calcium antagonists compared to ACEI/ARAII for severity, mortality and risk of intubation. Therefore, the use of ACEI/ARAII would be recommended as the first choice against calcium antagonists in COVID patients [65].

Regarding the use of diuretics in COVID-19, some studies claim that in-hospital use and initiation of diuretics during hospitalization, but not pre-hospital exposure, were associated with increased mortality in patients with COVID-19 [66]. Conversely, other studies have identified a protective association with thiazide diuretics, while ASA diuretics have been identified as a risk factor. In the analyzed literature, worse results in intubation, severity and mortality are observed in monotherapy than patients treated with ACEI/ARAII [57], and an improvement in patient data was observed when associating ACEI/ARAII with diuretics [66].

This study had some limitations. First, although all the selected studies analyzed the effect of antihypertensive drugs in patients with COVID-19, the great variability in study designs and characteristics results in heterogeneity of the results. In addition, the duration of the intervention and the different times at which the different parameters were measured can influence the results. A meta-analysis was performed, although it was not possible to include all the RCTs as there was great variability in the intervention programs. In addition, the follow-up of effects maintained over time was not analyzed. Therefore, it is necessary to conduct more RCTs with larger samples and examine the effects maintained over time.

Our results do not include some of the medium- and long-term benefits of appropriate use of antihypertensive drugs in patients who have suffered from COVID-19. The analysis of the expenses incurred per hospital stay, morbidity and mortality of these patients are interesting issues as recurrence of coronary events are sensitive problems that should be analyzed in future lines of research.

## 5. Conclusions

The findings suggest that the use of renin–angiotensin–aldosterone axis hypotensors should be maintained in the context of COVID-19 disease. In fact, a heterogeneous management of treatments other than ACEI and ARAII seems to worsen the prognosis in hospitalized patients.

Although there is no evidence of a clear role for the use of angiotensin blockers as antihypertensive treatment, their use does not harm patients with COVID-19. Furthermore, the use of other antihypertensive has not shown better results compared to ACEII and ARAII.

Because of this, it is advisable to follow the clinical guidelines and statements of International Scientific Societies that advise not to modify the use of ACEI/ARAII in patients with COVID-19.

## Figures and Tables

**Table 2:** Risk of bias of cohort studies using CASP critical reading guidelines.

Study	Question 1	Question 2	Question 3
Bean et al., 2020 [29]	Yes	Yes	Yes
De Spiegeleer et al., 2020 [31]	Yes	Yes	Yes
Elabd et al., 2021 [32]	Yes	Yes	Yes
Fosbϕl et al., 2020 [33]	Yes	Yes	Yes
Hippisley-Cox et al., 2020 [34]	Yes	Yes	Yes
Jeffery et al., 2022 [35]	Yes	Yes	Yes
Mancia et al., 2020 [37]	Yes	Yes	Yes
Mazzoni et al., 2022 [38]	Yes	Yes	Yes
Morales et al., 2021 [39]	Yes	Yes	Yes
Palazzuoli et al., 2020 [40]	Yes	Yes	Yes
Peñalvo et al., 2021 [41]	Yes	Yes	Yes
Sha et al., 2020 [42]	Yes	Yes	Yes
Trifiró et al., 2020 [44]	Yes	Yes	Yes
Zhang et al., 2020 [45]	Yes	Yes	Yes

Note: Question 1: Is the study focused on a clearly defined topic?; Question 2: Was the cohort recruited in the most appropriate way?; Question 3: Was the outcome measured precisely in order to minimize potential bias?

## Data Availability

Not applicable.
